# Essential Oil Composition of *Alluaudia procera* and in Vitro Biological Activity on Two Drug-Resistant Models

**DOI:** 10.3390/molecules24162871

**Published:** 2019-08-07

**Authors:** Paola Poma, Manuela Labbozzetta, Pietro Zito, Rosa Alduina, Aro Vonjy Ramarosandratana, Maurizio Bruno, Sergio Rosselli, Maurizio Sajeva, Monica Notarbartolo

**Affiliations:** 1 Department of Biological, Chemical and Pharmaceutical Science and Technology (STEBICEF), University of Palermo, Viale delle Scienze, 90128 Palermo, Italy; 2 Department of Agricultural, Food and Forest Sciences, University of Palermo Viale delle Scienze, 90128 Palermo, Italy; 3 Department of Plant Biology and Ecology, University of Antananarivo, PO Box 906, Antananarivo 101, Madagascar

**Keywords:** Didiereaceae, essential oil, acute myeloid leukemia cell, succulent plants

## Abstract

Drug resistance is a major obstacle in antibiotic and antitumor chemotherapy. In response to the necessity to find new therapeutic strategies, plant secondary metabolites including essential oils (EOs) may represent one of the best sources. EOs in plants act as constitutive defenses against biotic and abiotic stress, and they play an important role in the pharmacology for their low toxicity, good pharmacokinetic and multitarget activity. In this context, natural products such as EOs are one of the most important sources of drugs used in pharmaceutical therapeutics. The aim of this paper was to identify the chemical composition of the essential oil of *Alluaudia procera* leaves, obtained by hydrodistillation and analysed by gas chromatography-mass spectrometry, and to verify its biological activities on acute myeloid leukemia cancer cell HL60 and its multidrugresistant variant HL60R and the Gram-positive *Staphylococcus aureus* exhibiting multi-antibiotic resistance. We speculate that cytotoxic and antibiotic effects observed in the tested resistant models may be due to the coordinate activities of forty compounds detected or to the C_16_ macrocyclic lactones which are the major ones (30%). Our data confirm the possibility of using EOs as therapeutic strategies in resistant models is due to the heterogeneous composition of the oils themselves.

## 1. Introduction

**Figure 1:**
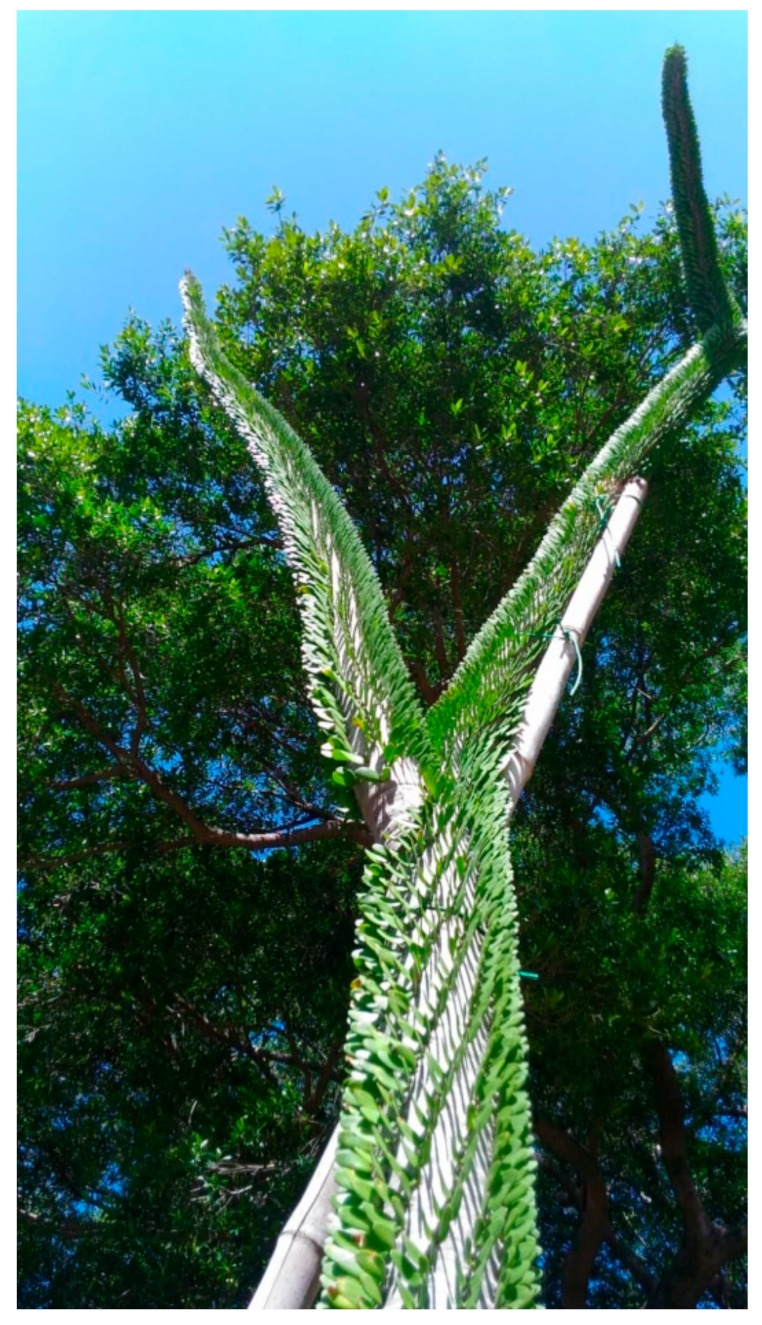
*Alluaudia procera* growing at the Botanical Garden of the University of Palermo.

Family Didiereaceae Drake includes six genera of xerophytic shrubs or small trees. The family is closely related to Cactaceae. The genus *Alluaudia* Drake is endemic to Southern Madagascar dry forest and composed of six species which are spiny trees usually 3 to 10 m [1]. *Alluaudia* spp. especially *A. ascendens* and *A. procera* are dominant trees in forest stands providing several ecosystem services. Abundant scientific literature reported their consumption by folivorous lemurs to supply protein and non-protein energy nutrient, fiber and water [2,3,4] but also their use as sleeping sites [5]. *Alluaudia procera* (Figure 1) is among extracted forest products used as fuel wood, making house and furniture [6,7].

*A. procera* has also some cultural value for local communities as the species is planted around burial sites to reinforce the taboos that protect them and also planted as a symbol of friendship or alliance [8]. Linholm et al. [9] investigated the cytotoxicity of a fractionated polypeptide fraction of *Alluaudia humbertii* which, among 100 species tested, showed a very low activity against human embryonal lung cells. Rasamoelisendra et al. [10] investigated the chemical composition of *Alluaudia dumosa* by methanol extraction. To the best of our knowledge, no data on the chemical composition of the essential oils (EOs) of the genus are available. The biological properties of EOs are widely documented from a vast literature that demonstrates in vitro anticancer, antibioceptive, antiviral, antiphlogistic and antimicrobial activity of these mixtures of volatile compounds [11,12]. This wide spectrum of biological activities is generally accompanied by biodegradability and reduced toxicity [12]. In plants, EOs act as constitutive defenses against biotic (e.g., herbivores and microorganisms) and abiotic (e.g., heath and light) stresses [13]. Succulent plants are adapted to dry environments where both environmental and biological stresses are high, and strong constitutive defenses are necessary to survive in their hostile habitat. For this reason, our team is investigating the EO composition of several species [14] of succulent plants to verify their possible useful biological activities against pathologies characterized by drug resistance. Multidrug resistance (MDR) is a major obstacle to successfully treating acute myeloid leukemia (AML) patients with chemotherapy. There is emerging evidence that the inability of the cells to undergo apoptosis contributes in several ways to the genesis and progression of cancer and may also represent a critical cause of tumor drug resistance. MDR is characterized by decreased intracellular drug accumulation after a period of drug administration, which is mediated by increase of drug efflux by ATP-binding cassette (ABC) transporters such as P-glycoprotein (P-gp) and multidrug resistance protein 1 (MRP1) [15,16,17]. Therefore, the search for novel anticancer drugs that are effective on multidrug resistant AML is in progress. In this context, natural products are one of the most important sources of drugs used in pharmaceutical therapeutics. Pharmacological resistance is represented also by the infectious drug resistant diseases. Microorganisms are present worldwide and the control of their growth and diffusion is of extreme importance in various environment, i.e., to combat infectious diseases in healthcare and in veterinary [18,19], or in cultural heritages to reduce biodeterioration processes [20]. Mulani et al. [21] indicated different therapeutic strategies against six nosocomial pathogens (ESKAPE) that exhibit multidrug resistance and virulence: *Enterococcus faecium*, *Staphylococcus aureus*, *Klebsiella pneumoniae*, *Acinetobacter baumannii*, *Pseudomonas aeruginosa* and *Enterobacter* spp. Persistent use of antibiotics has provoked the emergence of MDR and extensively drug resistant (XDR) bacteria, which render even the most effective drugs ineffective. The development of novel therapeutics to treat drug resistant infections could be represented by alternative therapies such as use of antibiotics in combination or with adjuvants, bacteriophages, antimicrobial peptides, nanoparticles and photodynamic light therapy but also by use of EOs [22]. In this paper we identified and tested the in vitro biological activities of the EO of *Alluaudia procera* in HL60 leukemia cell line and its MDR variant HL60R and on Gram-negative *Escherichia coli* and the Gram-positive *Staphylococcus aureus* exhibiting antibiotic resistance. 

## 2. Results

### 2.1. Chemical Composition

**Figure 2:**
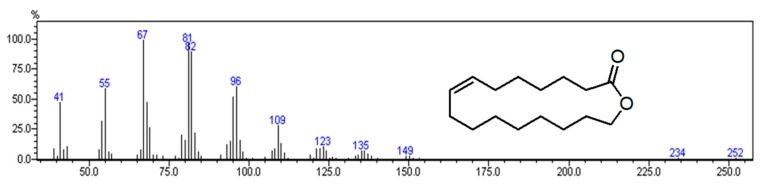
Mass spectrum and chemical structure of (*Z*)-Oxacycloheptadec-8-en-2-one (LRI 1914–2385) found in the present study.

In the EO of *A. procera* leaves forty compounds, representing about the 96% of the oil, were identified: Thirteen aliphatic hydrocarbons (9.3%), four oxygenated monoterpenes (0.8%), two sesquiterpene hydrocarbons (0.8%), two oxygenated sesquiterpenes (1.3%), three diterpene hydrocarbons (10.4%), one oxygenated diterpene (8.0%), six C_13_ norisoprenoids (5.4%) and seven macrolactones (58.7%) as shown in Table 1. The sample yielded 17.36 mg (0.003%).

(*Z*)-Oxacycloheptadec-8-en-2-one (18.1%) (Figure 2) with two (15.2% and 10.8%) of its four isomers were the most abundant compounds, followed by *trans*-phytol (8.0%), the third ambrettolide isomer (7.4%), neophytadiene (5.6%), the fourth ambrettolide isomer (3.8%) and vitispirane (3.7%). 

(*Z*)-Oxacycloheptadec-8-en-2-one is reported in the literature with several names and we report here those found in NIST [23]: Ambrettolid; Ambrettolide; Musk ambrette; Musk ambrette natural; Musk natural; Natural musk ambrette; Oxacycloheptadec-8-en-2-one, (*Z*)-; (*Z*)-7-Hexadecen-16-olide; 16-Hydroxy-7-hexadecenoic acid lactone, *cis*-; Oxacycloheptadec-8-en-2-one, (8*Z*)-; hexadec-7-en-16-olide. For a matter of simplicity, we will use the synonym ambrettolide through the text.

### 2.2. In Vitro Anticancer Activity

**Figure 3:**
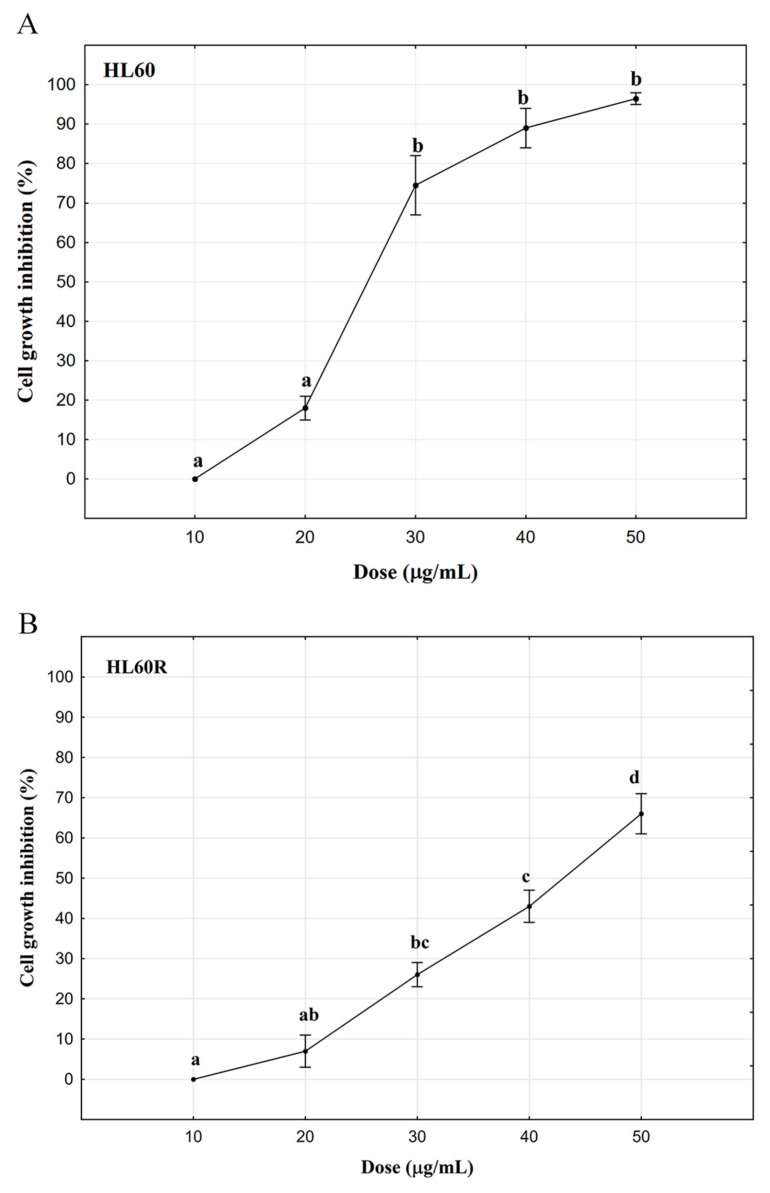
Cytotoxic activity of *A. procera* essential oil on HL60 (**A**) and HL60 R (**B**) cell lines. Cell viability was assessed by MTS. Data are expressed as mean ± standard error (SE) of at least three different experiments performed in triplicate. Different letters represent significant differences in cytotoxic activity among the concentrations of each cell line (Tukey test, *p* < 0.05).

The characteristics of the acute myeloid leukemia cell line HL60 and of its doxorubicin-resistant and MDR variant HL60R have been described previously [15]. Resistance of HL60R cell line were evaluated after two exposition steps to doxorubicin (250 ng in 8 mL of RPMI) by the trypan blue dye exclusion test. The cytotoxicity of *A. procera* EO in cancer cells has never been investigated. Under our experimental conditions, the EO induced a concentration-dependent reduction of cancer cell viability (Figure 3A,B) of HL60 and HL60R cells after 72 h of treatment. The concentration of essential oil that decreased cell viability to 50% (IC_50_) was 25.5 ± 5.5 μg/mL and 45.8 ± 6.2 μg/mL respectively. We co-treated same cell lines with EO in combination with doxorubicin, a conventional antiblastic drug often used as a first line treatment in the leukemia. As can be deduced from the data reported in Table 2, the expected percentage of cell viability is similar to that observed. These results indicate that the combination of EO and doxorubicin determines only an additive effect.

### 2.3. In Vitro Antibacterial Activity

**Figure 4:**
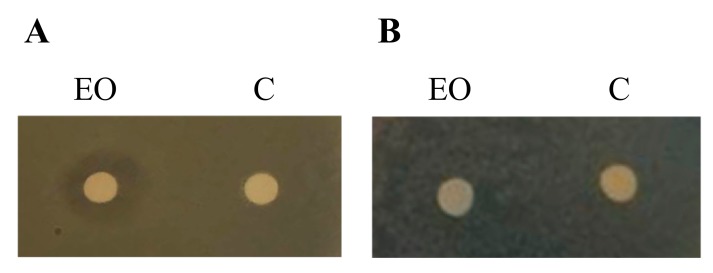
Antibacterial activity of the essential oil (EO) against *S. aureus* ATCC25923 (**A**) and *E. coli* K12 (**B**). The presence of the halo around the disk, previously soaked with the EO, demonstrates the antibacterial activity against *S. aureus* ATCC25923. C: Negative control.

The antibacterial activity of the EO was evaluated against the Gram-negative *E. coli* K12 and the Gram-positive *S. aureus* ATCC25923 by disc diffusion antibiotic sensitivity assays. The growth of the Gram-positive strain *S. aureus* was inhibited in the presence of 20 µL of EO at the concentration of 2 mg/mL, while the growth of *E. coli* was not (Figure 4). 

**Figure 5:**
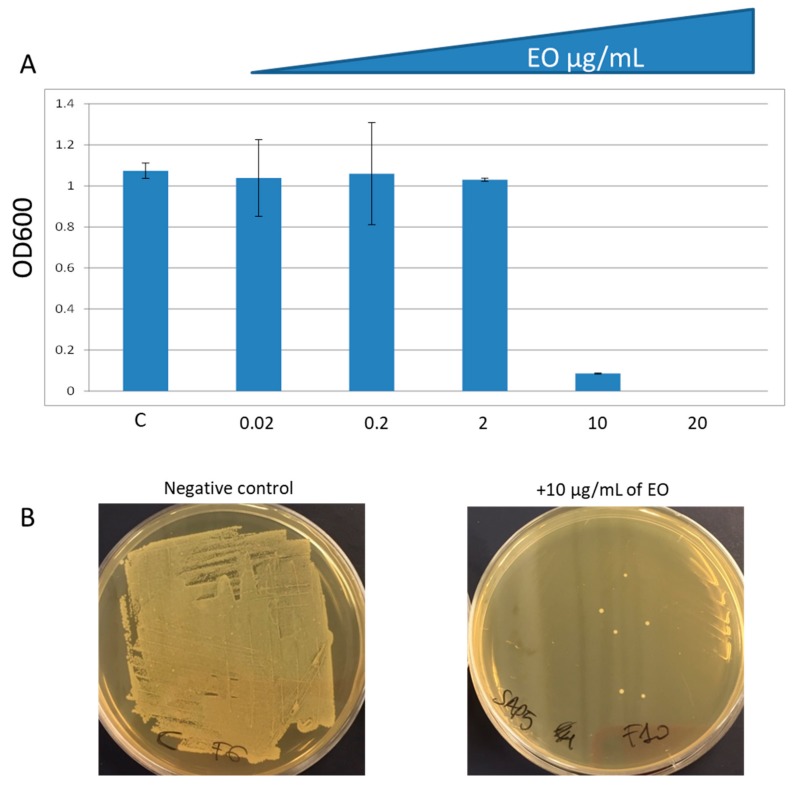
Effect of EO on *S. aureus* ATCC25923 growth. **A**: determination of total bacterial count. Data are average from triplicate experiments. Bars represent standard deviations of triplicate incubations. **B**: determination of vital bacterial count when 10 μg/mL of EO were added. Negative control represents the bacterial culture incubated with DMSO.

Increasing concentrations of EO were added to *S. aureus* ATCC25923 cultures to determine the minimal inhibitory concentration (MIC) by broth microdilution method. Evaluation of total bacterial count showed that the MIC of EO is 10 µg/mL, indeed the OD600 measurements were close to zero (Figure 5A). Thus, we plated a 100 µL aliquot of the bacterial suspension from the control well, and the well containing 10 and 20 µg/mL of EO onto the non-selective growth solid medium. The evaluation of vital bacterial cells showed that only 6 colonies grew in presence of 10 µg/mL EO (Figure 5B) and no colonies in presence of 20 µg/mL (data not shown). This latter result demonstrated that EO does not only inhibit bacterial growth but has a bactericidal effect and revealed that 20 µg/mL correspond to the minimal bactericidal concentration (MBC) (Figure 5B).

**Figure 6:**
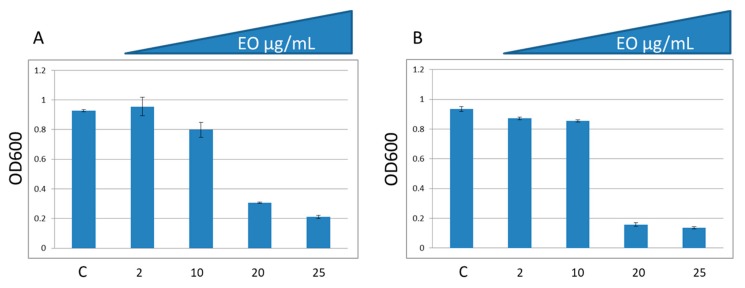
Effect of EO on growth of two MDR *S. aureus* isolates (**A**—*S. aureus* strain 11; **B**—*S. aureus* strain 5). Data are average from triplicate experiments. Bars represent standard deviations of triplicate incubations.

The addition of 25 µg/mL of EO inhibited the growth of two MDR clinical isolates (*S. aureus* strain 5 and 11) which are resistant to penicillin, erythromycin, oxacillin, cephalosporins and amoxicillin-clavulanic acid. (Figure 6). 

The comparison of the antibacterial activity of EO, erythromycin (Ery) and the combination of EO+Ery against the MDR *S. aureus* isolate 11 indicated that when erythromycin was used in combination with EO the effect was due to EO only (Table 3). 

## 3. Discussion

The aim of this paper was to identify the chemical composition of the EO of *A. procera* leaves and to verify its biological activity in two acute myeloid leukemia cell lines and against two microorganisms, *E. coli* and *S. aureus*. The chemical composition of the EO is quite peculiar. It includes higher terpenes (i.e., diterpenes), whereas mono and sesquiterpene are almost absent. Phytol, neophytadiene and its isomers constitute the terpenic fraction of the oil. The occurrence of C_13_ norisoprenoids is very common in plants. In fact, edulans, vitispirane, trimethyl-dihydronaphthalenes, very flavouring volatile substances, are often found together contributing to the overall fruit flavour; also, *trans*-β-damascenone is one of the most potent of all known wine flavor compounds. All of them (C_13_ norisoprenoids) derive from different carotenoid degradation pathways, and this fact confirms the plants’ disposition towards diterpenes biosynthesis. They, besides having several biological effects, contribute to constitutive defenses of the plant [24]. Interestingly, the major constituents of essential oil are five C_16_ macrocyclic lactones that together constitute 58.7% of the EO composition. Ambrettolide [(*Z*)-oxacycloheptadec-8-en-2-one] is the major component (18.1%) along with four isomers, similarly to Leffingwell et al. [25] in Latakia tobacco volatiles. Ambrettolide is a valuable flavor material isolated from ambrette (*Abelmoschus moschatus* Medicus = *Hibiscus abelmoschus* L.) seeds where it is the second most abundant compound (13%) [26]. This compound is rare among plants [27], and it has been found, 0.7% to 1.9%, in the headspace of the flowers of two tropical orchids [28] and in the essential oil (0.8%) of the flowers of *Trollius europaeus* (Ranunculaceae) [27].

The GC-MS analysis of *A. procera* EO showed the presence of five main peaks identified by the libraries as ambrettolide with a similarity ranging from 90% to 96%. A comparison with the linear retention indices (LRI) reported in literature allowed us to identify the major of these peaks as ambrettolide. Ambrettolide (LRI 1914 - 2385) was verified by authentic standard as reported in Materials and Methods. The remaining were attributed to positional and/or configurational isomers of the double bond that could arise biogenetically or by isolation procedure (hydrodistillation and thermal isomerization).

Other two compounds, eluting after the ambrettolide and their isomers, have been matched as ambrettolide by the libraries. As reported by other authors [29,30] for the oils of *Hibiscus abelmoschus* (= *Abelmoschus moschatus*), another macrolide homologue of ambrettolide has been identified as oxacyclononadec-10-en-2-one. It has been reported by Leffingwell et al. [25] also. On the basis of these findings, we have assigned the structures of oxacyclononadec-10-en-2-one to one of these two compounds occurring in *A. procera* leaves EO and its isomeric form to the other one, similarly as for the ambrettolide, comparing the LRI reported in literature. Their MS spectra [30] are very similar to ambrettolide, and this can explain the mismatched identification by the libraries. The analysis of *A. procera* leaves EO showed that it is mainly (58.7%) composed by macrolactones (macrolides) conferring to it powerful biological activity. The presence of a high percentage of macrolides may contribute to the constitutive defense of *A. procera* leaves against possible microorganism attack.

The presence of macrolides in *Alluaudia* may probably contribute to the defense against microorganisms preventing rooting of the leaves. The pharmacological profile of *A. procera* EO is very interesting probably because of the high percentage content of macrolide compounds. Macrolides contain macrocyclic lactone ring and constitute a wide family of natural products, many of which present antibiotic activity. The non-antibiotics macrolide drugs like tacrolimus, everolimus and sirolimus are immunosuppressants, used in transplant rejection and in the treatment of some autoimmune diseases, while everolimus and sirolimus are used as immunosuppressants and antitumor drugs [31]. Despite having different pharmacological activities, macrolide compounds are substrate of P-gp [32], in particular the antitumor effects of everolimus and sirolimus are well documented as inhibitors of cellular proliferation through mTOR (mammalian target of rapamycin) inhibition in some tumor models such as renal cell carcinoma, hepatocellular carcinoma and breast cancer [33,34]. For all these reasons, we investigated both the antitumor and antimicrobial activities of *A. procera* EO in two resistant models. We compared the biological effect of EO in the acute myeloid leukemia cell line HL60 with its MDR variant HL60R that in contrast to their parental cells lacked sensitivity to cell death induction from diverse stimuli, including doxorubicin and cisplatin administration [35]. Drug resistance in HL60R variant cells is characterized by overexpression of a variety of proteins, which belong to the P-gp but also by constitutive expression of inhibitor of apoptosis proteins (IAPs), family members proteins that we previously described play an important role in tumor cell resistance to drug induced apoptosis and show also a different expression in drug-resistant and -sensitive HL60 leukemia cells [35,36]. Consistent with the biological role of IAPs, it has been reported that they may adversely affect the prognosis of patients with solid tumors or acute myeloid leukemia [37,38]. Screening of several natural products in the search for novel anticancer agents against human leukemia HL60 and its multidrug resistant variant HL60R cells led us to identify antitumor activity in the EO from *A. procera* leaves. The different cytotoxic level of EO between HL60 and HL60R, like doxorubicin, could indicate that C_16_ macrocyclic lactones could be substrate of P-gp. Since the antitumor drug doxorubicin still today is the first-choice treatment for AML in combination with cytarabine and/or etoposide [38], there is an urgent need to explore alternative therapeutic approaches to improve outcomes. In our cellular model, which consist of a cell line doxorubicin resistant, we carried out cytotoxicity assay using a combination of essential oil of *A. procera* and doxorubucin. As this combination causes only an additive cytotoxic effect, we suppose that essential oil or its major compounds are substrate of P-gp, as indeed many macrolides are. It is known that doxorubicin administered in increasing doses is able to enhance the transcription of the gene that encodes for P-gp via NF-κB, making the cells resistant to chemotherapy [35]. The mechanism of action of the C_16_ macrocyclic lactones found in the present study is unknown, but on the basis of our results, we think that they could exert an antiproliferative effect by inhibiting mTOR and/or NF-κB like structural analogues, substrate of P-gp used as anticancer agents. The presence of C_16_ macrocyclic lactones also led us to verify the potential effect on resistant bacteria. In the last years, the appearance of multidrug-resistant microorganisms has increased. Therefore, there is the urgent necessity to find new, safe, efficient and economical compounds [39,40,41] for fighting the spread of multidrug resistant bacteria, especially in healthcare.

Our results show that the EO is active against *S. aureus*. Interestingly the EO was active also against the MDR clinical isolates tested, which are resistant to penicillin, erythromycin, oxacillin, cephalosporins and amoxicillin-clavulanic acid. The clinical isolates were characterized by the coordinate expression of some genes that encode for endotoxins and mecA gene expression (methicillin resistant), which confer an aggressive phenotype [42,43]. Our results show that EO exerts a bacteriostatic and bactericidal effects against *S. aureus*, unlike macrolide antibiotic compounds which are bactericidal only at high doses. We surmise that EO works inside the Gram-positive cells thanks to its lipophilic properties. The EO showed no effect against *E. coli* since the protection due to the LPS layer could prevent its entry. It is interesting to note that when supplying EO in combination with erythromycin the antimicrobial effect is due to the EO only.

The chemical composition of oil derived from the seeds of *Abelmoschus moschatus* L. (Malvaceae), named ambrette seed oil, showed ambrettolide as the second major compound [26]. In this paper, the authors suggested that the antimicrobial effect of ambrette seed oil may be due to damages in the cell membranes because of its hydrophobicity [26]. The authors stated that the Gram-positive bacteria are more sensitive to essential oils than Gram-negative bacteria because of the higher charged outer membrane. 

Our data indicate that *A. procera* EO induced cytotoxic effect in the cell lines investigated and also as antimicrobial against the *S. aureus* isolates. Both models show MDR and the efficacy of EO may be further tested to verify possible pharmacological applications. Further studies are necessary to verify if the biological activities described are due to the major compounds, namely ambrettolide, or to a combination of the identified compounds. In conclusion, although this is a new and emerging area of cancer research, the ability of EOs and their components to have such diverse antiproliferative activities through acting on various pathways and cellular mechanisms is engaging. Moreover, the possibility of using essential oils as therapeutic strategies in resistant models is due to the heterogeneous composition of the oils themselves; for this reason, the propensity to develop drug resistance is unlikely different from what happens when using conventional drugs consisting of a single molecule.

## 4. Materials and Methods

### 4.1. Plant Species

*Alluaudia procera* Drake is a small tree branching from near the base with spiral rows of spines [44,45]. At the start of every growing season leaves are produced in pairs above the spines and fall during the dry season [1]. *A. procera* is listed in CITES Appendix II together with the whole family Didiereaceae. Its seeds are exempt from the provisions of CITES. The IUCN red list reports this species as near threatened [46].

### 4.2. Plant Material

Leaves of *Alluaudia procera* were collected in July 2018 from plants cultivated at the Botanical Garden of the University of Palermo. The plants were raised from seeds in 1984 and cultivated in the open with reference code: Didiereaceae 4. The seeds were obtained and the plants raised before the Convention on Biological Diversity (CBD) entered into force on 29 December 1993 and therefore are pre-CBD specimens. The matrices were placed in paper bags and kept at −30 °C for 24 h before hydrodistillation. No specific permits were required for the described location and for the collection of plant material because the plants are part of the living collection of the Botanical Garden of the University of Palermo and the authors have access to that. 

### 4.3. Essential Oil Isolation and Chemical Characterization

The matrices *A. procera* (600 g—about 1700 leaves) were hand cut in small pieces and hydrodistillated for 3 h in a Clevenger-type apparatus, using *n*-pentane as collection solvent [47]. The oil was dried by anhydrous sodium sulphate and stored at −30 °C until chemical analysis and pharmacological tests. To prepare the stock solution for biological studies 2 mg of essential oil were dissolved in 1 mL of dimethyl sulfoxide (DMSO). The GC analysis was performed in an Agilent 7000C GC system (Agilent, Santa Clara, CA, USA), fitted with a fused silica Agilent HP-5MS capillary column (30 m × 0.25 mm i.d.; 0.25 μm film thickness, ), coupled to an Agilent triple quadrupole Mass Selective Detector MSD 5973; ionization voltage 70 eV; electron multiplier energy 2000 V; transfer line temperature, 295 °C. Helium was the carrier gas (1 mL min^−1^). The other GC analysis was performed in a Shimadzu QP 2010 plus (Shimadzu, Kyoto, Japan), single quadrupole GC/MS system, fitted with a Supelcowax 10 capillary column (30 m × 0.25 mm i.d.; 0.25 μm film thickness, (Merck KGaA, Darmstadt, Germany); ionization voltage 70 eV; transfer line temperature, 280 °C. Helium was the carrier gas (1 mL min^−1^). For both columns, the temperature was initially kept at 40 °C for 5 min, then gradually increased to 250 °C at 2 °C min^−1^ rate, held for 15 min and finally raised to 270 °C at 10 °C min^−1^. One μL of diluted samples (1/100 *v*/*v*, in *n*-pentane) was injected at 250 °C automatically and in the splitless mode; transfer line temperature, 295 °C.

### 4.4. Identification of Compounds

Identification of compounds was carried out using NIST 11, Wiley 9, FFNSC 2, and Adams [48] databases. These identifications were confirmed by Linear Retention Indices (LRI) with those available in literature by SciFinder database. Some of the compounds were also confirmed by comparison of mass spectra and retention times with standard compounds available in laboratory. (*Z*)-Oxacycloheptadec-8-en-2-one authentic standard (100 μg/mL in cyclohexane), product code DRE-XA15756500CY, was purchased by LGC DR Ehrenstorfer^TM^. The retention indices were determined in relation to a homologous series of *n*-alkanes (C_8_–C_30_) injected under the same operating conditions. Component relative (%) amounts were calculated based on GC peak areas without using correction factors.

### 4.5. Cell Culture

The HL60 cells were obtained from ATCC^®^ (CCL-240, Rockville, MD, USA), while its variant HL60R, was selected for multidrug resistance (MDR) by exposure to gradually increasing concentrations of doxorubicin. One milliliter of cell suspension was distributed into each well of 24-well culture plates and 24 h later doxorubicin was added. After 24 h cell viability was determined by the ability of cells to exclude trypan blue dye and expressed as percent of control cell growth. The cells were routinely maintained in RPMI-1640 (HyClone Europe Ltd., Cramlington, UK) supplemented with 10% heat-inactivated fetal calf serum, 1% l-glutamine, 1% penicillin/streptomycin solution (all from HyClone Europe). The cells were grown in a humidified atmosphere at 37 °C in 5% CO_2_. Cells having a narrow range of passage number were used for all experiments. The cultured were routinely tested for *Mycoplasma* infection.

### 4.6. Cytotoxicity Assay

Exponentially growing cells were suspended at 5 × 10^4^ cells/mL in complete medium, and 200 μL of cell suspension were distributed into each well of 96-well microtiter plates and incubated overnight at 37 °C. At time 0, the medium was replaced with fresh complete medium supplemented of essential oil at the indicated concentrations. Following 72 h of treatment, 16 μL of a commercial solution (obtained from Promega Corporation Madison, WI, USA) containing 3-(4,5-dimethylthiazol-2-yl)-5-(3-carboxymethoxyphenyl)-2-(4-sulphophenyl)-2*H*-tetrazolium (MTS) and phenazine ethosulfate were added. The plates were incubated for 4 h in a humidified atmosphere at 37 °C in 5% CO_2_. The bioreduction of the MTS dye was assessed by measuring the absorbance of each well at 490 nm. Cytotoxicity was expressed as a percentage (mean ± SE) of the absorbance measured in the control cells.

### 4.7. Microorganisms

Two reference strains, *Staphylococcus aureus* ATCC25923 and *Escherichia coli* K12, and two toxigenic and multidrug resistant (MDR) *S. aureus* strains 11 and 5 isolated from clinical environments [42] were used in this study and were maintained as described elsewhere [43].

### 4.8. Determination of the Antibacterial Activity

The antibacterial activity was assessed against the reference strains, *S. aureus* ATCC25923 and *E. coli* K12, by using a disc diffusion antibiotic sensitivity assays as described in literature [49,50]. In brief, a dense suspension (∼10^7^ cells) of each microorganism was spread onto the Luria Bertani agar medium (LB-agar; 10 g/L Tryptone, 5 g/L yeast extract, 10 g/L NaCl, 18 g/L Bacto agar, pH 7.2). Steril paper discs of Whatman filter paper (No. 42) of uniform diameter (0.6 cm) were used to spot directly different volumes of EO, EO+Ery or Ery, as described in the figure legend. Then, the discs were placed on the overlay of bacteria on LB-agar plate. Growth inhibition halos were observed after overnight incubation at 37 °C. The antibacterial activity was considered if an inhibition halo of growth larger than 0.6 cm (size of the disk paper) was produced. The antibacterial activity was calculated at least as a mean of three replicates.

The antibacterial activity against the MDR *S. aureus* strains (11 and 5) was assessed by the broth microdilution method; briefly, bacteria were incubated at 37 °C in a Luria Bertani (LB) broth (10 g/L tryptone, 10 g/L NaCl, 5 g/L Yeast extract, pH 7.2). After overnight growth, a 1:100 dilution of the bacterial suspension was incubated at 37 °C in a microplate containing LB medium and increasing concentrations of *A. procera* EO (from 0 to 25 µg/µL). Total bacterial concentration was measured at the time of the inoculum and after 18h of incubation by reading the optical density at wavelenght of 600 nm using a microplate reader (GloMax^®^ - Multi Detection System). The experiment was performed in triplicate and the average optical density was calculated. After growth in liquid medium, 100 µL of bacterial suspension incubated in presence of different amounts of EO was spread on LB agar plates, that were incubated at 37 °C overnight. After incubation, the colonies were counted to evaluate the viable bacterial count. As a control, bacterial suspension was incubated in the presence of DMSO, solvent in which EO was dissolved.

### 4.9. Statistical Analysis

Results of bioassays are given as means ± standard error (SE). Statistical analysis was carried out according to Poma et al. [4] by analysis of variance (one-way ANOVA) followed by Tukey’s test. Statistica ver. 12 (StatSoft Inc. 1984–2014) was used as software for the analyses.

## Figures and Tables

**Table 1:** Chemical composition of *A. procera* essential oil.

LRI ^a^	LRI ^b^	Compound	%	Id ^c^	Class ^d^
1069	1435	*cis*-Linalool oxide	0.2	1, 2	OM
1085	1436	*trans*-Linalool oxide	0.1	1, 2	OM
1099	1551	Linalool	0.2	1, 2, 3	OM
1186	1693	α-Terpineol	0.3	1, 2, 3	OM
1253	1463	*cis*-Edulan	0.3	1, 2	C13
1272	1508	Vitispirane (isomer not identified)	3.7	1, 2	C13
1309	1595	*trans*-Edulan	0.9	1, 2	C13
1345	1734	1,1,6-Trimethyl-1,2-dihydronaphthalene	0.2	1, 2	C13
1380	1807	*trans*-β-Damascenone	0.1	1, 2	C13
1383	1804	1,4,6-trimethyl-1,2-dihydronaphthalene	0.2	1, 2	C13
1411	1569	*cis*-*α*-Bergamotene	0.5	1, 2	SH
1508	1735	(*E*,*E*)-α-Farnesene	0.3	1, 2	SH
1634	2032	1-*epi*-Cubenol	0.3	1, 2	OS
1700	1700	Heptadecane	0.2	1, 2, 3	H
1738	2492	γ-Costol	1.0	1, 2	OS
1756	2606	Benzyl benzoate	0.5	1, 2, 3	O
1800	1800	Octadecane	0.4	1, 2, 3	H
1838	1926	Neophytadiene (isomer not identified)	5.6	1, 2	DH
1863	1955	Neophytadiene (isomer not identified)	1.1	1, 2	DH
1880	1982	Neophytadiene (isomer not identified)	3.7	1, 2	DH
1900	1900	Nonadecane	0.9	1, 2, 3	H
1906	2418	Ambrettolide isomer	7.4	1, 2	ML
1914	2385	Ambrettolide [(*Z*)-Oxacycloheptadec-8-en-2-one]	18.1	1, 2, 3	ML
1919	2414	Ambrettolide isomer	15.2	1, 2	ML
1924	2373	Ambrettolide isomer	10.8	1, 2	ML
1928	2393	Ambrettolide isomer	3.8	1, 2	ML
2000	2000	Eicosane	0.5	1, 2, 3	H
2100	2100	Heneicosane	0.6	1, 2, 3	H
2111	2614	*trans*-Phytol	8.0	1, 2, 3	OD
2114	2586	14-Methyl-8-hexadecyn-1-ol	1.2	1, 2	O
2119	2575	Oxacyclononadec-10-en-2-one (isomer not identified)	1.3	1, 2	ML
2128	2610	Oxacyclononadec-10-en-2-one (isomer not identified)	2.1	1, 2	ML
2200	2200	Docosane	0.3	1, 2, 3	H
2300	2300	Tricosane	0.4	1, 2, 3	H
2400	2400	Tetracosane	0.1	1, 2, 3	H
2500	2500	Pentacosane	0.8	1, 2, 3	H
2600	2600	Hexacosane	0.5	1, 2, 3	H
2700	2700	Heptacosane	3.1	1, 2, 3	H
2800	2800	Octacosane	0.4	1, 2, 3	H
2900	2900	Nonacosane	1.1	1, 2, 3	H
		**Class of Compounds**			
		Oxygenated Monoterpene	0.8		
		Sesquiterpene Hydrocarbons	0.8		
		Oxygenated Sesquiterpene	1.3		
		Diterpene Hydrocarbons	10.4		
		Oxygenated Diterpene	8.0		
		Aliphatic Hydrocarbons	9.3		
		C_13_ Norisoprenoids	5.4		
		Macrolactones	58.7		
		Others	1.7		
		Total	96.4		

^a^ Linear Retention Index on a HP-5 MS column; ^b^ linear Retention Index on a Supelcowax 10 column; ^c^ 1: retention index; 2: MS, mass spectrum; 3: co-injection with authentic compound; ^d^ OM: oxygenated monoterpenes, SH: sesquiterpene hydrocarbons, OS: oxygenated sesquiterpenes, DH: diterpene hydrocarbons, H: hydrocarbons, C13: C_13_ norisoprenoids, ML: macrolactones, O: others.

**Table 2:** Cell growth inhibition in HL60 and its multidrug resistant variant HL60R following treatment with essential oil of *A. procera* and doxorubicin (Doxo) or a combination of these.

Cell Lines and Treatments	Cell Viability (%)	Expected (%)
**HL60**		
Essential oil of *A. procera* 10 μg/mL	100.0 ± 0.0	
Essential oil of *A. procera* 20 μg/mL	82.0 ± 2.1	
Doxo 1 ng/mL	90.0 ± 0.7	
Doxo 2 ng/mL	84.5 ± 3.9	
Essential oil of *A. procera* 10 μg/mL + Doxo 1ng/mL	100.0 ± 0.5	90.0 ± 0.7
Essential oil of *A. procera* 10 μg/mL + Doxo 2 ng/mL	93.0 ± 3.1	84.5 ± 3.9
Essential oil of *A. procera* 20 μg/mL + Doxo 1 ng/mL	80.0 ± 1.7	74.0 ± 1.4
Essential oil of *A. procera* 20 μg/mL + Doxo 2 ng/mL	71.0 ± 2.2	69.0 ± 1.4
**HL60R**		
Essential oil of *A. procera* 10 μg/mL	100.0 ± 0.0	
Essential oil of *A. procera* 20 μg/mL	70.5 ± 3.2	
Doxo 100 ng/mL	87.5 ± 3.9	
Doxo 500 ng/mL	76.5 ± 4.6	
Essential oil of *A. procera* 10 μg/mL + Doxo 100 ng/mL	95.0 ± 2.2	87.5 ± 3.9
Essential oil of *A. procera* 10 μg/mL + Doxo 500 ng/mL	88.0 ± 3.8	76.5 ± 4.6
Essential oil of *A. procera* 20 μg/mL + Doxo 100 ng/mL	70.0 ± 1.9	62.0 ± 5.6
Essential oil of *A. procera* 20 μg/mL + Doxo 500 ng/mL	61.0 ± 5.9	54.0 ± 5.6

Data are expressed as the mean ± standard error of three independent experiments. There are not statistical differences among expected (%) and cell viability (%). Expected value: Sum of the effects of the agents alone minus that of the untreated cells.

**Table 3:** Results of the disc diffusion antibiotic sensitivity assays. The diameter of the inhibition halos around the disk, previously soaked with EO and EO+Ery, is reported.

Incubation of Bacteria in Presence of	Diameter (cm) of the Inhibition Halo
EO	0.8 ± 0.05
EO + Ery	0.8 ± 0.03
Ery	0 ± 0.01
